# Monkey upload: Improving robustness using multi-stage neural alignment

**DOI:** 10.1167/jov.26.6.1

**Published:** 2026-06-01

**Authors:** Utkarsh Jain, Shreya Sumbetla, Garrison W. Cottrell

**Affiliations:** 1 Department of Computer Science and Engineering, University of California, San Diego, La Jolla, CA, USA; 2 Department of Computer Science and Engineering, University of California, San Diego, La Jolla, CA, USA; 3 Department of Computer Science and Engineering, University of California, San Diego, La Jolla, CA, USA

**Keywords:** convolutional neural networks, neural alignment, primate visual system

## Abstract

Recent research has shown that aligning neural network representations with neural recordings has the effect of regularizing them (Federer, Xu, Fyshe, & Zylberberg, 2020; Li et al., 2019; Pirlot, Gerum, Efird, Zylberberg, & Fyshe, 2022), improving their performance across several metrics. However, previous work in this field has predominantly used neural data from the V1 cortex, and comparable performance gains are obtained when the V1 data are replaced by a noise distribution with similar statistics (Federer et al., 2020; Pirlot et al., 2022). In this research, we present the results of aligning the hidden layers of a convolutional neural network model with macaque brain recordings from V1, V4, and IT. Previous research, using only data from the inferior temporal cortex (Dapello et al., 2022), resulted in improved robustness to adversarial examples. Here we show that aligning all three areas improves the model’s robustness against several different kinds of corruption. Furthermore, we show that the standard alignment approach, deep canonical correlation analysis (DCCA) is not necessary to achieve good results. We found that when using the VICReg unsupervised loss function for alignment, the model displays more reliable robustness against a subset of corruptions than DCCA. When aligning all three areas, VICreg is superior to InfoNCE (Oord, Li, & Vinyals, 2019) and DCCA, which show robustness to 4, 2, and 0 distortions, respectively. Finally, we show that DCCA is very sensitive to randomizing the data, VICReg is mildly affected, and InfoNCE is not sensitive at all. Hence, for two of the models, it is not just the distribution of the data that matters when aligning all three areas. Overall, our research provides further support for the impact of neural data in developing more robust and neurobiologically plausible models of vision.

## Introduction

Deep convolutional neural networks (CNNs) have made remarkable progress in recent years, surpassing human-level performance on several tasks. Although originally inspired by the primate visual system, CNNs lack the adaptability to novel inputs inherent in human vision (Szegedy et al., 2014; Nguyen, Yosinski, & Clune, 2015; Geirhos et al., 2018; Hendrycks & Dietterich, 2019). This gap between CNNs and human performance underscores the differences between the mechanisms used by the human visual system and those of computer vision models, and raises concerns about the deployment of neural networks in real-world noisy environments where safety is paramount. One potential solution to this problem is to better align neural networks with the primate visual system.

In this article, we constrain the representations learned by the computer vision model to be more aligned with those in the macaque visual system to transfer meaningful inductive biases from the macaque brain. Previous work (Li et al., 2019; Federer, Xu, Fyshe, & Zylberberg, 2020; Safarani et al., 2021; Pirlot, Gerum, Efird, Zylberberg, & Fyshe, 2022) in this direction has explored co-training networks to force their early-stage representations to V1 representations from the primate brain. These studies primarily report an improvement in downstream task performance and increased robustness against adversarial attacks and common corruptions. However, the V1 cortex is known to encode Gabor-like filters, which respond only to simple features like edges and bars. This factor makes the V1 cortex highly sensitive to the slightest of changes in the input image, making V1 representations very noisy and unstable. As a result, these studies have also observed a comparable improvement in performance when the actual data are replaced with a noise distribution with similar statistics (Federer et al., 2020).

More recently, Dapello et al. (2022) investigated the effect of aligning late-stage CNN representations with inferior temporal (IT) data and observed a significant improvement in the model’s human behavioral alignment, along with similar improvements in robustness against adversarial attacks. Kietzmann et al. (2019) co-trained a network on the object categorization task with multi-stage - V1, V4, and IT-alignment with human magnetoencephalography (MEG) data and compared the performance of feedforward and recurrent networks in capturing the activation dynamics of the visual stream.

Although the previous work investigated robustness to adversarial attacks, in this article, we extend this work by investigating the effect of multi-stage neural alignment (V1, V4, and IT) on robustness to the 19 noise types that have been used in previous benchmarking work (Hendrycks & Dietterich, 2019; Safarani et al., 2021). We hypothesized that aligning multiple stages of the network with the corresponding neural responses should yield better results compared with aligning a single stage alone. To test this hypothesis, we experiment with constraining the V1, V4, and IT-like layers in the CORnet-Z model (Kubilius et al., 2019) with neural data from the corresponding stages in the visual ventral stream of macaque monkeys. We find that this treatment does perform better than single-stage alignment for a subset of perturbations.

Furthermore, previous research predominantly used sophisticated statistical loss functions, such as DeepCCA (Pirlot et al., 2022) and centered kernel alignment (Dapello et al., 2022), to enhance the alignment between the model’s hidden representations and brain representations. Here, we study the effect of substituting these loss functions with simple contrastive loss functions and analyze their performance. We show that the VICReg contrastive loss function (Bardes, Ponce, & LeCun, 2021) is superior to DCCA in providing robustness to distortions. This finding demonstrates that simpler loss functions can effectively improve corruption robustness when using multi-stage alignment.

### Related work

Yamins et al. (2014) demonstrated that, as CNNs improve at the object categorization task, their internal representations become increasingly predictive of the neural activity in the primate brain. Specifically, they showed that later-stage representations in CNNs were the most predictive of IT neural responses, whereas representations from mid-level stages were most predictive of V4 neural responses. This established a strong model-layer-to-brain-layer correspondence between CNNs and the primate visual system. Building on these results, Sinz, Pitkow, Reimer, Bethge, and Tolias (2019) introduced the *neural co-training* hypothesis, suggesting that co-training a neural network on a downstream task and a neural response prediction task could bias the network into learning useful and robust representations from neurophysiological recordings, thus bringing them closer to human performance.

Li et al. (2019) were among the first to test this hypothesis. They co-trained ResNet-18 on two losses–the cross-entropy loss from the CIFAR-10 image classification task and a representation mismatch loss based on the mean squared error between the neural and model representational similarity matrices (Kriegeskorte, Mur, & Bandettini, 2008). By aligning the V1-like layer of ResNet-18 with mouse V1 neural data generated from a predictor model, they reported an improvement in the model’s robustness against random noise and adversarial attacks. Federer et al. (2020) used a similar multi-task training setup with CORnet-Z (Kubilius et al., 2019) and the CIFAR-100 dataset, aligning the V1 layer of the model with the V1 neural dataset collected by Coen-Cagli, Kohn, and Schwartz (2015). They observed an increase in the object categorization accuracy and robustness to label corruption, even when the actual neural data were replaced with randomly generated data with similar statistics or the data with the targets shuffled. Furthermore, these improvements are observed only when V1 data are used to regularize the early layers of the CNN model. When used to regularize mid-level and later layers, the model performed worse than the baseline, further solidifying the model-to-brain correspondence.

Extending upon Li et al. (2019), Safarani et al. (2021) trained a VGG-19 network on the Tiny ImageNet classification task with V1 neural alignment and observed an increase in the model’s robustness against 9 of the 14 corruptions from the TinyImageNet-C dataset (Hendrycks & Dietterich, 2019), with a steady increase in the robustness as more neural data was introduced during the training. Following Federer et al. (2020), Pirlot et al. (2022) experimented with deep canonical correlation (Andrew, Arora, Bilmes, & Livescu, 2013) loss and reported even greater improvements in results across the same metrics. More recently, Dapello et al. (2022) aligned the later stages of the CORnet-S model (Kubilius et al., 2019) with neural data from the IT cortex, reporting an improvement in the IT alignment on held-out animal and image sets, enhanced adversarial robustness, and better alignment with human behavior on several benchmarks.

## Methods

Here we describe the image and neural datasets, alignment loss functions, the training methods for multi-stage alignment, and methods for assessing the network’s robustness against corruptions.

### Data

#### Image data

For the object categorization task, we use the CIFAR-100 dataset (Krizhevsky 2009), which contains 100 classes with 50,000 training images and 10,000 test images. We hold out 10% of the training dataset (50 images per class; 5,000 images in total) as a validation set. Training images are augmented with 32×32 random cropping with 4 pixels of padding and random horizontal flipping, then normalized using standard CIFAR-100 image statistics. Testing and validation images are normalized in the same way. To evaluate the network’s robustness to image noise, we used the CIFAR-100-C dataset (Hendrycks & Dietterich, 2019), which includes 19 different types of corruptions with 5 levels of severity for each, emulating varying intensities of real-world perturbations. These images are normalized with the standard CIFAR-100 testing set statistics and are used solely for testing the network’s corruption robustness. We emphasize here that we do not augment the training set with the corruption statistics to ensure the network does not encounter distortions during training.

#### Neural data

Aligning the network at multiple stages requires data from the corresponding regions in the primate visual ventral stream, based on the findings from prior work (Yamins et al., 2014; Kietzmann et al., 2019; Federer et al., 2020). We use V1 neurophysiological recordings collected by Cadena et al. (2019) from two rhesus macaque monkeys, comprising 7,250 image–response pairs from 166 neurons. For V4 and IT data, we use the recordings collected by Majaj, Hong, Solomon, and DiCarlo (2015), which include responses for 3,200 images from 88 neural sites in the V4 cortex and 168 sites in the IT cortex. The neural data are used exclusively for training the network, with no portion held out for validation or testing. The network is regularized with the neural data for the entirety of the training.

### Alignment loss

Following Pirlot et al. (2022), we used deep canonical correlation analysis (DCCA) (Andrew et al., 2013) to align the network and brain representations. Additionally, we explore the efficacy of contrastive loss functions for neural alignment. Below, we briefly describe the different loss functions used in our research.

#### DCCA

DCCA is an extension of canonical correlation analysis (CCA) (Hotelling, 1936; Anderson, 1984) that aims to maximize the correlation between two views of data. CCA finds coefficient vectors $a_{1}^{*}$ and $b_{1}^{*}$ that maximize the correlation between the two different views of the data, *x*, *y* :

(1) $$\begin{array}{c} a_{1}^{*},b_{1}^{*}=\underset{a,b}{Argmax}\;\mathrm{cov}\left(a^{⊤}x,b^{⊤}y\right),\; \end{array}$$

where (*a*^⊤^*x*, *b*^⊤^*y*) is called the first canonical pair. The subsequent pairs of coefficient vectors are found by identifying vectors that optimize Equation (1) and are orthonormal to all previous pairs of coefficient vectors. The total number of canonical pairs found is controlled with hyperparameter *C*. The relationship between these two views, *x* and *y*, is described by the set of *C* canonical correlations, indexed by *i*, between $\left(a_{i}^{⊤}x,b_{i}^{⊤}y\right)$ (Pirlot et al., 2022).

DCCA, unlike traditional CCA, nonlinearly transforms the two views through independent multi-layer perceptrons and then applies the usual CCA optimization procedure to align the two representations. As discussed by Andrew et al. (2013), and highlighted by Pirlot et al. (2022), DCCA effectively handles nonlinear relationships between two views of the data and can filter out noise in either view. For our purposes, the two views are the network and brain representations. The loss equation can be represented as:

(2) $$\begin{array}{c} L_{dcca}=-\mathrm{CCA}\left(f_{x}\left(X,\theta_{x}\right),f_{y}\left(Y,\theta_{y}\right)\right),\; \end{array}$$

where, *X* and *Y* represent the two views of the data for which we want to maximize the correlation; *f*_*x*_ and *f*_*y*_ represent the two independent multi-layer perceptrons; θ_*x*_ and θ_*y*_ represent the weights associated with *f*_*x*_ and *f*_*y*_; and  CCA  is the sum over all the canonical pairs’ correlation.

#### Information Noise-Contrastive Estimation (InfoNCE)

InfoNCE loss is a contrastive learning technique introduced in Oord, Li, and Vinyals (2019) and used primarily in self-supervised learning tasks. It encodes the two views in compact representations that maximally preserve the mutual information of the original signals. By doing so, we extract the underlying latent variables the inputs have in common. We used the NT-Xent loss implementation as proposed in Chen, Kornblith, Norouzi, and Hinton (2020). The loss expression maximizes the similarity between positive pairs and considers all the other examples in the minibatch as negative pairs. In our model, we maximized the similarity between the model’s representations for one image with its corresponding brain representations while taking all the other stimuli’s brain representations in the batch as negative samples. The loss equation for a positive pair of samples (*i*, *j*) can be expressed as:

(3) $$\begin{array}{c} L_{ince}=-\log\frac{\exp(\mathrm{sim}\left(z_{i},z_{j}\right)/\tau )}{\sum_{k=1}^{2N}1_{\left[k\neq i\right]}\exp\left(\mathrm{sim}\left(z_{i},z_{k}\right)/\tau \right)},\; \end{array}$$

where sim(*z*_*i*_, *z*_*j*_) represents the cosine similarity of the two representations *z*_*i*_ and *z*_*j*_, and τ denotes the temperature parameter. The final loss is aggregated across all positive pairs in a mini-batch.

#### Variance-Invariance-Covariance Regularization (VICReg)

VICReg (Bardes, Ponce, & LeCun, 2021) is a regularization technique designed to enhance the quality of learned representations in self-supervised learning tasks. The primary goal of VICReg is to encourage the learned representations to exhibit three key statistical properties—variance (maintained across data samples), invariance (minimized between the two views of data), and covariance (minimized between the different features of the same view). Hence, this helps to bring the model and neural representations closer (invariance term) while maintaining a varied and rich representation (variance term) and reducing redundancy within the features of each view (covariance term). The loss equations can be represented as:

(4) $$\begin{array}{ccc} & & \;L_{vicr}\left(Z,Z^{'}\right)=\alpha s\left(Z,Z^{'}\right)\\ & & \;+\mu \left[v\left(Z\right)+v\left(Z^{'}\right)\right]+\nu \left[c\left(Z\right)+c\left(Z^{'}\right)\right]\; \end{array}$$

(5) $$\begin{array}{ccc} & & \;s\left(Z,Z^{'}\right)=\frac{1}{n}\sum_{i}{∥z_{i}-z_{i}^{'}∥}_{2}^{2},\\ & & \;v\left(Z\right)=\frac{1}{d}\sum_{j=1}^{d}\max\left(0,\gamma -\sqrt{\mathrm{Var}(z_{j})+\varepsilon }\right)\; \end{array}$$

(6) $$\begin{array}{ccc} & & \;c\left(Z\right)=\frac{1}{d}\sum_{i\neq j}{\left[C\left(Z\right)\right]}_{ij}^{2},\\ & & \;C\left(Z\right)=\frac{1}{n-1}\sum_{i=1}^{n}\left(z_{i}-\bar{z}\right){\left(z_{i}-\bar{z}\right)}^{T},\\ & & \;where\;\bar{z}=\frac{1}{n}\sum_{i=1}^{n}z_{i}, \end{array}$$

where *Z* and *Z*′ are the two representations of the same data; *z*_*i*_ and $z_{i}^{'}$ are the embeddings for the *i*^*th*^ sample; *d* is the dimensionality of the representations; γ is a constant target value for the standard deviation; α, μ and ν are fixed weights for the weighted loss; and ϵ is a small scalar to improve numerical stability.

### “Monkey upload”: Neural co-training

**Figure 1.**
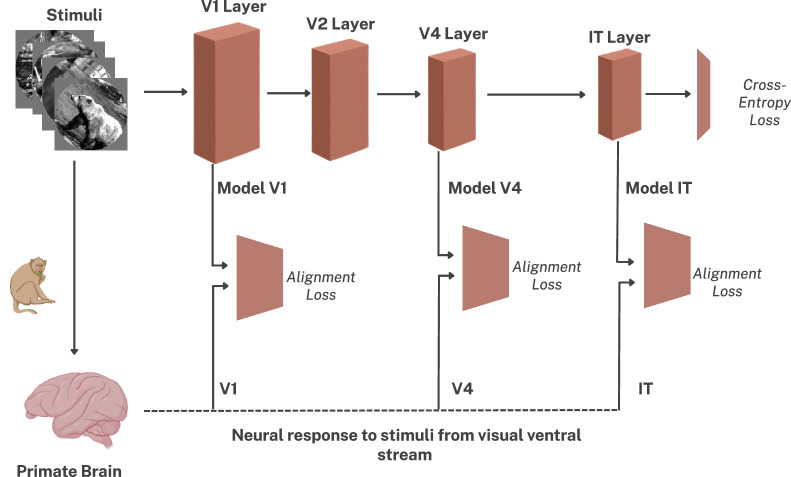
CORnet-Z architecture for multi-task training on the CIFAR-100 object categorization task and multi-stage neural alignment. The V1, V4, and IT layers of the model are aligned with the matching neural dataset with independent alignment loss modules. We experiment with three alignment losses—DCCA, VICReg and InfoNCE. The computed alignment loss backpropagates through the portion of the network used for obtaining the corresponding brain representations.

We used a co-training setup similar to that used by Pirlot et al. (2022) to train the CORnet-Z model on the CIFAR-100 object categorization task with V1, V4, and IT neural alignment (Figure 1). The network is trained on two losses—cross-entropy loss for the object categorization task and alignment loss for the multi-stage neural alignment. We selected CORnet-Z as our base CNN model because of its simple model-layer-to-brain-area correspondence, although our multi-stage neural alignment approach can be adapted to other CNN architectures as well.

Images from the CIFAR-100 training set are processed by the CORnet-Z model, and trained with cross-entropy loss, $L_{ce}$. The neural datasets consist of image stimuli shown to macaques and the corresponding neural recordings. To obtain the model representations, we process these images through the network and extract the feature maps from the matching computational blocks. These extracted feature maps are aligned using the various loss functions described above (see Alignment loss). Following the formulation used by Pirlot et al. (2022), we combine the four loss terms—$L_{ce}$, $L_{alignment}^{\left\{V1,V4,IT\right\}}$—using a slightly modified version of their equation (compare with Equation 8):

(7) $$\begin{array}{ccc} & & \;L_{total}=\sum_{block}\lambda_{block}\cdot L_{alignment}^{block}\\ & & \;+\left(1-\sum_{block}\lambda_{block}\right)\cdot L_{ce},\; \end{array}$$

where *block* ∈ {*V*1, *V*4, *IT*}, and λ_*block*_ controls the contribution of $L_{alignment}^{block}$ in the combined loss $L_{total}$. If λ_*block*_ is set to 0 for a particular block, the corresponding model representations are not aligned. We used the following loss, omitting the (1 − ∑_*block*_λ_*block*_) coefficient to make setting the λ’s simpler:

(8) $$\begin{array}{c} L_{total}=\sum_{block}\lambda_{block}\cdot L_{alignment}^{block}+L_{ce}.\; \end{array}$$

The revised formulation allows λ_*block*_ to take any positive real value. To keep our results consistent with previous work, we only experiment with λ_*block*_ ∈ {0, 0.1, 0.25, 0.5, 0.75, 0.9}. We define the *baseline model* by setting all λ_*block*_ values to zero, with only $L_{ce}$ contributing to the combined loss.

### Experimental setup

Our setup comprises four main components: CORnet-Z object categorization model, alignment module, CIFAR-100 dataset, and neural datasets from V1, V4, and the IT cortex. We use the official implementation of the CORnet-Z, but modified to include a 100-way classification head suitable for the CIFAR-100 dataset. The model is optimized on the combined loss with stochastic gradient descent, using a momentum of 0.9, a learning rate of 10^−3^, and an *L*_2_ weight decay of 10^−4^. All the models are run for 500 epochs, 10 times with 10 different seeds. To control for variations beyond the regularizer, when comparing models, we use the same seeds for each model so they all start with the same initial weights and the same order of data presentation. This strategy is strictly analogous to a within-subjects design in psychology, with the advantage that the subject is not affected by the other treatment. Because of the common set of stochastic factors used for both methods induced by the seed, the resulting evaluations are dependent rather than independent. We therefore test significance using paired *t*-tests on the seed-wise differences in performance. This makes the test sensitive to systematic performance differences between methods while avoiding inflation of variance that would arise from incorrectly treating matched runs as independent samples.

**Figure 2.**
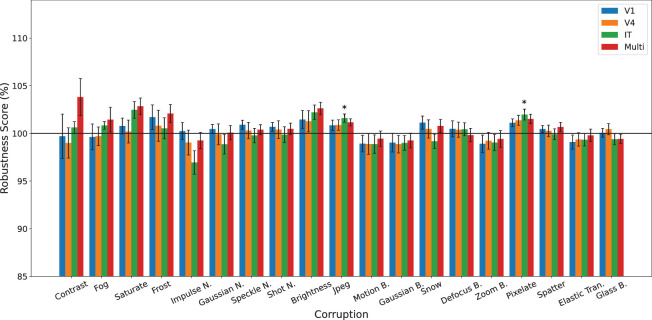
DCCA model comparison of multi-stage with single-stage V1, V4, and IT alignment on robustness to 19 CIFAR-100-C corruptions, averaged across five severity levels. The horizontal line at 100% corresponds with the baseline model. Colors denote different alignment choice: V1 blue; V4 orange; IT green; and all three simultaneously, denoted as “multi” in red. The only conditions showing statistically significant improvements over the baseline are IT-only alignment for JPEG compression and pixelate. Corruptions are sorted in descending order of relative gain of the multi-stage model over the baseline, computed across 10 seeds per model. Error bars denote ±1 standard error of the mean across 10 random seeds.

Our baseline is the CORnet-Z model trained using only the cross-entropy loss, without any regularization from neural recordings. We compare this baseline with models trained with alignment losses (DCCA, VICReg, and InfoNCE), while keeping the architecture and optimization settings identical. Performance is evaluated using the classification accuracy on 5 severity levels of the 19 corruptions in the CIFAR-100-C dataset to isolate the effect of the regularization strategy on robustness. We take the average of the accuracies across the five severity levels for each corruption. The robustness scores plotted in Figures 2–^3^,^4^,5 and, Supplementary Figure S2 represent the relative performance gain calculated as $\left(\frac{\text{avg}\_\text{model}\_\text{score}}{\text{avg}\_\text{baseline}\_\text{score}}\right)\times 100$, where values above 100% indicate improvement over the baseline. This normalization enables comparison across corruptions with differing baseline (no-regularization) performance levels, enabling a more interpretable visualization of relative gains. Accordingly, the figures include a horizontal line at 100%, denoting the baseline reference. As seen in Supplementary Figure S1, the final model accuracy on the CIFAR100 dataset is only around 45%, which is expected due to our use of the CORnet-Z architecture, a shallow convolutional neural network chosen to preserve compatibility with the primate visual system and enable direct neural alignment.

As in previous work, we train our neurally aligned model on λ_*block*_ ∈ {0.1, 0.25, 0.5, 0.75, 0.9} and we analyze the results of the best model (w.r.t. CIFAR-100 holdout set) on CIFAR-100-C. We used paired *t*-tests across matched runs for each corruption. We then corrected for multiple comparisons using false discovery rate (Benjamini–Hochberg, threshold of 0.05).

For the three regularization modules (marked “Alignment Loss” in Figure 1), we use the same setting as Pirlot et al. (2022) with each of the regularization sub-networks consisting of two independent 3 layered MLPs, each of width 1,024 with ReLU activation function, followed by a dropout layer, and a final fully connected layer of width 10. The weights of the loss modules are initialized from a normal distribution with a mean of zero and a standard deviation of 0.01 and that of the CORnet-Z model are initialized with Xavier Uniform initialization. We use RMSprop with a learning rate of 10^−4^ and an *L*_2_ weight decay of 10^−5^ to optimize the regularization modules. We use a batch size of 256 for the CIFAR-100 dataset and a batch size of 50 for all the neural datasets. The single-stage versus multi-stage alignment experiments in experiment 1 were conducted using the DCCA loss function. With the specified hyperparameters, each training epoch took around 14 seconds for the baseline model, 49 seconds for the single-stage aligned models, and 77 seconds for the multi-stage aligned models (averaged across the different loss functions).

For our experiments with other contrastive losses (InfoNCE and VICReg), we maintain all the experimental setup parameters (including identical seeds, which ensure the same weight initialization and data ordering) and test the model with the best λ_*block*_ validation accuracy. We use a temperature of 0.5 for InfoNCE loss and weights of (2, 2, 1) for the invariance, variance, and covariance components of the VICReg loss, with a variance epsilon of 10^−4^. We trained all our models on one machine with a single NVIDIA RTX A6000 GPU.

## Results

### Experiment 1: Comparison of multi-stage alignment versus single-stage on corruptions

We first investigated the different effects of single- versus multi-stage-alignment on the 19 corruptions using the DCCA objective, and compare their performance against the baseline (Figure 2). We expect multi-stage alignment to have the best results. Unexpectedly, given previous work, this was not the case. In fact, only two corruption types (pixelate and JPEG compression) show any significant performance over baseline, and only for the IT-only alignment. As far as we know, this exact evaluation (CIFAR-100-C and CORNet-Z) has not been done before, so it is difficult to compare this result with previous work.

Given the error bars in Figure 2, for example on multi-stage alignment (red bars) for contrast, fog, saturate, frost, and brightness, it may be puzzling why they are not significant. The answer is that these differences did not survive correction for multiple comparisons. See Supplementary Table S1 for complete statistics for these, before and after correction.

### Experiment 2: Contrastive losses yield more reliable robustness than DCCA

**Figure 3.**
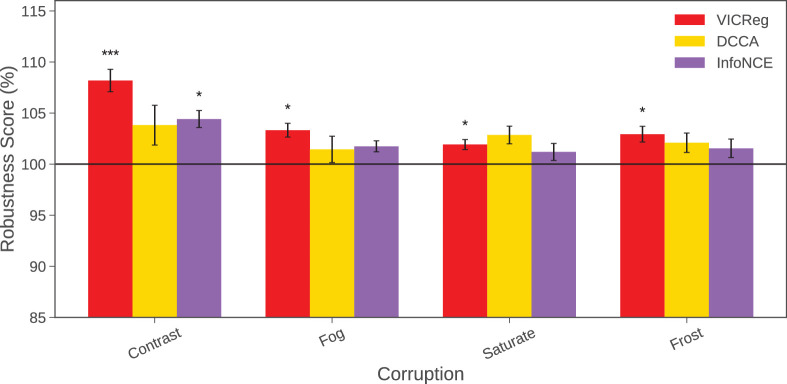
Results for the four corruptions scores vs. baseline (the 100% line) for which there are statistically significant results. These are all multi-stage alignment models (DCCA data taken from Figure 2). Error bars denote ±1 standard error of the mean computed across 10 random seeds. Asterisks indicate a significant difference from the baseline score (*p < 0.05, **p < 0.01, ***p < 0.001).

In this experiment, we compare the performance of all three objective functions with respect to the baseline (not with each other^1^) on multi-stage alignment, as we hypothesize that these can lead to more reliable robustness results. Across experiments, the alignment module was varied, while all other model parameters and training settings were held constant to ensure a fair comparison. We then compared each model’s performance on the 19 corruptions with respect to the baseline. We performed paired *t*-tests across matched runs for each corruption, applying false discovery rate (Benjamini–Hochberg) correction to account for multiple comparisons with a significance threshold of 0.05. In Figure 3, we show the results for the four corruptions that gave statistically significant results. VICReg demonstrates the most reliable robustness gains over the baseline (the 100% line), with statistically significant improvements for appearance-based distortions: contrast, *t*(9) = 7.2194, *p* = 0.00005, *f*_*FDR*_ = 0.00095, and saturation, *t*(9) = 3.6953, *p* = 0.005, *f*_*FDR*_ = 0.023; as well as environmental distortions: fog, *t*(9) = 4.7301, *p* = 0.001, *p*_*FDR*_ = 0.01, and frost, *t*(9) = 3.7103, *p* = 0.005, *p*_*FDR*_ = 0.023.

For completeness, Supplementary Figure S1 shows the average performance in all 19 corruptions and the 5 severity levels for each of the loss functions tested. Although VICReg achieves numerically higher robustness scores across a broad range of additional corruptions, none of these are even significant before correction (Supplementary Table S2). Training speed across the three models are comparable, with DCCA being numerically, but not significantly, the slowest.

In contrast, InfoNCE yields limited statistically reliable robustness gains, with significance observed only for contrast, *t*(9) = 5.0486, *p* = 0.0007, *p*_*FDR*_ = 0.013, (see Figure 3), while improvements for other corruptions appear as numerical trends that do not survive correction. Surprisingly, DCCA does not produce *any* statistically significant robustness improvements under paired testing after correction. We attribute this to our setup: CorNet-Z and CIFAR-100, which we do not believe has been tested with DCCA. This indicates that VICReg’s regularization influence is better suited for learning representations resilient to at least some real-world corruptions.

### Experiment 3: Comparing performance with permuted data

Previous work has shown that regularization applied to V1 gives similar results to just using permuted data, suggesting that only the distribution of the data matters. This makes sense, as V1’s representations should shift a great deal in the face of small translations, that is, V1 is not translation invariant. To test whether recordings from higher-level areas, where representations are known to be somewhat translation invariant, influence the model performances, we ran a control experiment where the neural activity pattern was randomly permuted. Differences here will measure each objective function’s sensitivity to the structure in the data, not just the distribution.

**Figure 4.**
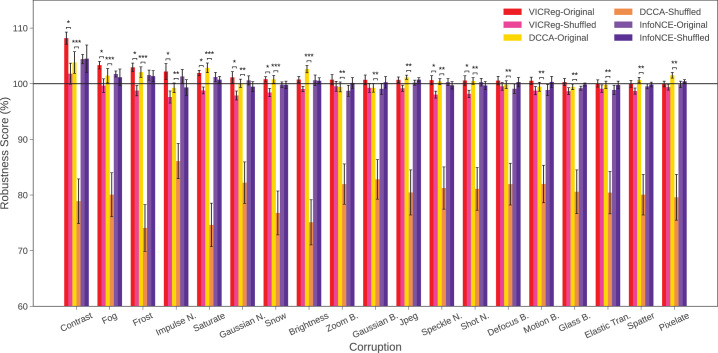
Robustness scores of control experiment when trained with shuffled data for each of the contrastive loss functions. Error bars denote ±1 standard error of the mean computed across 10 random seeds. Asterisks indicate a statistically significant difference between the model and corresponding shuffled experiment for a particular loss function (*p < 0.05, **p < 0.01, ***p < 0.001).

**Figure 5.**
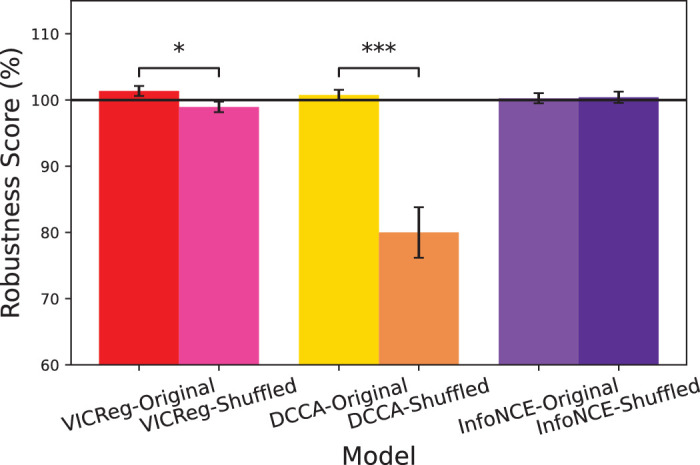
Average robustness scores for correct data–target pairing versus shuffled data for each of the contrastive loss functions (averaged across all corruptions). Error bars denote ±1 standard error of the mean computed across 10 random seeds. Asterisks indicate a statistically significant difference between the model and corresponding shuffled experiment for a particular loss function (*p < 0.05, **p < 0.01, ***p < 0.001).

The detailed results of this experiment are given in Figure 4. We observe that shuffling the neural data leads to huge drops in robustness across *all* corruptions for DCCA (yellow vs. orange bars), suggesting it is highly sensitive to the structure of the data. The results for VICReg (red vs. pinkish bars) are less consistent, but show statistically significant differences between shuffled and control for 9 out of 19 corruptions (*p* < 0.05), including contrast, fog, frost, and several noise and weather-related perturbations. In contrast, InfoNCE is not impacted at all by shuffling the data. We summarize these results in Figure 5, by averaging across the 19 corruptions. As expected from the detailed results, both DCCA, *t*(9) = 5.6421, *p* = 0.0003, and VICReg, *t*(9) = 3.2198, *p* = 0.01, have significantly better performance than their corresponding shuffled models.

Overall, these results confirm that the robustness gains observed with VICReg are not explained by random regularization alone, but reflect meaningful contributions from neural alignment.

## Discussion and conclusions

In this work, our primary objective was to build on prior research by exploring the benefits of multi-stage alignment using V1, V4, and IT neural data. Our findings differ from previous results in that DCCA, even when applied to all three levels of representation, does not lead to improvements in robustness to corrupted data.

This is curious, especially in the face of the results that DCCA is extremely sensitive to the structure of the data (experiment 3), and the results of Dapello et al. (2022). However, there are large differences in methodology. First, they used the CKA instead of DCCA. Second, they used a CorNet-S architecture (much deeper than CorNet-Z, the smallest CorNet architecture), that was pretrained on ImageNet, which would give rise to a much richer feature space than CIFAR-100. Third, for alignment, they presented their monkeys with artificially generated single-object images (on a plain background) from 10 categories, such as bear, elephant, face, and so on, suggesting a lower-dimensional overall manifold of stimuli. This may have had a stronger regularization effect in those strategy categories due to its relative homogeneity, presumably evoking more consistent neural responses from monkey cortex.

Our results in experiment 1 support our initial hypothesis that using V1 neural data is not as effective as higher-level alignment. We hypothesize that V1 recordings will tend to be more variable due to the V1 cortex’s high sensitivity to slight changes in fixations, and aligning later stages, especially concurrently, would yield greater benefits. In addition to this, we also wanted to test the efficacy of simple contrastive loss functions with respect to the sophisticated similarity indices often used in previous work.

The underlying motivation for our research was to test if we can make neural networks more robust without explicitly training them on different corruptions. Although it is possible to train the network directly on corruptions, previous work has shown that the effectiveness of this technique does not generalize to corruptions not covered during training. Aligning models with neural representations serves as a better solution in this regard because neural co-training has been shown to generalize the model against different corruptions without seeing them during training. We believe our results suggest the need for larger neural datasets that can be used to make the model more brain-aligned.

One potential improvement to our model would be to use a *predictor model*, similar to Safarani et al. (2021) or Li et al. (2019), to learn stimulus-to-response mapping and use that model to synthetically generate a larger neural dataset. This technique has shown promising results (Li et al., 2019; Safarani et al., 2021) and we believe it could serve as an effective solution until bigger neural datasets are collected and made available. Finally, we would like to test the benefits of multi-stage alignment with more neurobiologically plausible architectures such as LocRNN (Veerabadran, Ravishankar, Tang, Raina, & Sa, 2023) and CORnet-S (Kubilius et al., 2019). We aim to compare their performance against a wider benchmark to further validate the efficacy of our approach.

## Supplementary Material

Supplement 1

## Footnotes

1 Comparisons between models on individual corruptions did not lead to any interesting significant results.
