# Supplementary material for: Monkey upload: Improving robustness using multi-stage neural alignment
Source: J Vis. 2026 Jun 1;26(6):1. doi: 10.1167/jov.26.6.1 (PMC13235757; doi:10.1167/jov.26.6.1)
Supplement: Supplement 1 [file jovi-26-6-1_s001.pdf]

Supplementary Material for Monkey Upload: Improving Robustness using Multi-stage Neural Alignment

Utkarsh Jain
Department of Computer Science and Engineering
University of California, San Diego, La Jolla, California, USA

Shreya Sumbetla
Department of Computer Science and Engineering
University of California, San Diego, La Jolla, California, USA

Garrison W. Cottrell
Department of Computer Science and Engineering
University of California, San Diego, La Jolla, California, USA

Appendix A

Main Significance Test Results

This section highlights the p values and confidence intervals for the statistically significant results across all experiments. The detailed tables for each model are presented in Appendix B.

Table S1: Statistically significant robustness improvements for each loss function and alignment stage (paired t-tests across seeds). Multi-stage implies neural-alignment with all 3 visual layers (V1, V4 and IT). The Single-stage experiments are all run with DCCA loss function. Bold p-values indicate raw significance ($p < 0.05$). Full results are reported in Appendix B.

Loss / Stage	Corruption	Diff	95% CI	p	p_{FDR}
VICReg Loss (Multi-stage)	contrast	2.0128	[1.3821, 2.6435]	**0.00005**	**0.000946**
	fog	1.0338	[0.5394, 1.5282]	**0.00107**	**0.010201**
	frost	0.9426	[0.3679, 1.5173]	**0.00484**	**0.023543**
	saturate	0.5590	[0.2168, 0.9012]	**0.00496**	**0.023543**
InfoNCE Loss (Multi-stage)	contrast	1.0848	[0.5987, 1.5709]	**0.00069**	**0.013141**
	fog	0.5384	[0.1398, 0.9370]	**0.01367**	0.129814
DCCA Loss (Multi-stage)	brightness	1.0072	[0.4081, 1.6063]	**0.00420**	0.079727
	jpeg_compression	0.4970	[0.1103, 0.8837]	**0.01739**	0.089861
	pixelate	0.6700	[0.1392, 1.2008]	**0.01892**	0.089861
	saturate	0.8318	[0.2288, 1.4348]	**0.01231**	0.089861
V4 (DCCA Loss)	pixelate	0.6104	[0.1301, 1.0908]	**0.018987**	0.360752
IT (DCCA Loss)	impulse_noise	-1.2062	[-2.0889, -0.3235]	**0.013573**	0.064549
	jpeg_compression	0.6371	[0.2821, 0.9922]	**0.003263**	**0.045377**
	pixelate	0.8804	[0.3551, 1.4058]	**0.004777**	**0.045377**
	saturate	0.6807	[0.1824, 1.1789]	**0.013589**	0.064549

Note. Diff = mean(Neural-aligned model – baseline (no regularization)). p values are two-tailed paired t-tests across seeds. p_{FDR} denotes Benjamini–Hochberg adjusted p across corruption types.

doi: Received: April 24, 2026 ISSN 1534–7362 © 2024 ARVO

Appendix B

Detailed Significance Test Results

This appendix contains the full statistical results for all robustness comparisons. Specifically, we include significance tables for multi-stage alignment using VICReg (Table S2), InfoNCE (Table S3), and DCCA (Table S4), and for single-stage alignment at V1 (Table S5), V4 (Table S6), and IT (Table S7) (all trained with the DCCA loss), with all conditions evaluated relative to the baseline (no regularization).

Loss Comparison Detailed Graphs

Figure S1 shows performance across severity levels for InfoNCE, VICReg, DCCA, and the baseline. Figure S2 reports robustness across all 19 corruptions with paired t-test results between across models.

Table S2: Paired t-tests comparing VICReg (multi-stage neural alignment) vs. baseline average robustness accuracies across 10 seeds for each corruption type. Reported are mean±SD per condition, mean difference (Neural-aligned–baseline) with 95% CI, t(df), two-tailed p and Benjamini–Hochberg FDR-adjusted p. Bold indicates $p < 0.05$.

Corruption	n	Baseline (Mean±SD)	Neural-aligned (Mean±SD)	Diff [95% CI]	t(df)	p	p_{FDR}
brightness	10	38.5704±0.6132	38.8538±0.7708	0.2834 [–0.2233, 0.7901]	1.2653(9)	0.237526	0.577827
contrast	10	24.7328±0.5121	26.7456±0.6605	2.0128 [1.3821, 2.6435]	7.2194(9)	**0.000050**	**0.000946**
defocus_blur	10	39.3360±0.6529	39.5528±1.0570	0.2168 [–0.5011, 0.9347]	0.6831(9)	0.511721	0.694479
elastic_transform	10	39.6832±0.6459	39.7006±0.9928	0.0174 [–0.5946, 0.6294]	0.0643(9)	0.950126	0.950126
fog	10	31.1314±0.3560	32.1652±0.7561	1.0338 [0.5394, 1.5282]	4.7301(9)	**0.001074**	**0.010201**
frost	10	32.4812±0.6698	33.4238±0.6477	0.9426 [0.3679, 1.5173]	3.7103(9)	**0.004843**	**0.023543**
gaussian_blur	10	36.8574±0.7569	37.1044±1.0898	0.2470 [–0.5181, 1.0121]	0.7303(9)	0.483805	0.694479
gaussian_noise	10	38.3990±0.8211	38.8090±0.6264	0.4100 [–0.5554, 1.3754]	0.9607(9)	0.361787	0.694479
glass_blur	10	40.9016±0.3473	41.0224±0.7643	0.1208 [–0.4935, 0.7351]	0.4449(9)	0.666931	0.791981
impulse_noise	10	31.5040±0.7879	32.1634±0.8253	0.6594 [–0.4100, 1.7288]	1.3948(9)	0.196532	0.577827
jpeg_compression	10	43.0866±0.3449	43.3790±0.6570	0.2924 [–0.2373, 0.8221]	1.2486(9)	0.243296	0.577827
motion_blur	10	35.6704±0.8610	35.8522±1.0363	0.1818 [–0.3700, 0.7336]	0.7452(9)	0.475132	0.694479
pixelate	10	44.4638±0.3772	44.4382±0.6778	–0.0256 [–0.5689, 0.5177]	–0.1066(9)	0.917454	0.950126
saturate	10	29.3134±0.4581	29.8724±0.4435	0.5590 [0.2168, 0.9012]	3.6953(9)	**0.004957**	**0.023543**
shot_noise	10	40.5094±0.7147	40.7234±0.5876	0.2140 [–0.6229, 1.0509]	0.5784(9)	0.577158	0.731067
snow	10	36.2324±0.5059	36.5176±0.4315	0.2852 [–0.1171, 0.6875]	1.6038(9)	0.143220	0.544234
spatter	10	39.1806±0.3955	39.1622±0.5450	–0.0184 [–0.5939, 0.5571]	–0.0723(9)	0.943923	0.950126
speckle_noise	10	40.2052±0.6375	40.4442±0.5798	0.2390 [–0.5390, 1.0170]	0.6949(9)	0.504662	0.694479
zoom_blur	10	37.6016±0.8617	37.8660±1.2698	0.2644 [–0.5494, 1.0782]	0.7350(9)	0.481073	0.694479

Note. Diff = Neural-aligned–baseline. p values are two-tailed; p_{FDR} denotes Benjamini–Hochberg adjusted p across corruption types.

Figure S1: Performance on 19 corruptions from CIFAR-100-C with multi-stage neural alignment comparing the 3 different contrastive loss functions: DCCA, InfoNCE, and VICReg.

Figure S2: Robustness score comparison between DCCA, InfoNCE, and VICReg Loss for multi-stage alignment model. Among the pairwise significance tests between loss functions, only DCCA outperforms InfoNCE on pixelate after FDR correction. The corruptions are sorted in the descending order of relative gain of the multi-stage VICReg aligned model with respect to the baseline across 10 seeds per model. Error bars denote ±1 standard error of the mean (SEM) computed across 10 random seeds.

Table S3: Paired t-tests comparing InfoNCE (multi-stage neural alignment) vs. baseline (no regularization) average robustness accuracies across $n = 10$ seeds for each corruption type. Reported are mean±SD per condition, mean difference (Neural-aligned–baseline) with 95% CI, t(df), two-tailed p, Benjamini–Hochberg FDR-adjusted p.

Corruption	n	Baseline (Mean±SD)	Neural-aligned (Mean±SD)	Diff [95% CI]	t(df)	p	p_{FDR}
brightness	10	38.5704±0.6132	38.7930±0.8242	0.2226 [–0.6576, 1.1028]	0.5721(9)	0.581282	0.782240
contrast	10	24.7328±0.5121	25.8176±0.6266	1.0848 [0.5987, 1.5709]	5.0486(9)	**0.000692**	**0.013141**
defocus_blur	10	39.3360±0.6529	38.9624±0.7296	–0.3736 [–1.1855, 0.4383]	–1.0409(9)	0.325069	0.595361
elastic_transform	10	39.6832±0.6459	39.2250±0.7513	–0.4582 [–1.2600, 0.3436]	–1.2927(9)	0.228317	0.595361
fog	10	31.1314±0.3560	31.6698±0.4305	0.5384 [0.1398, 0.9370]	3.0558(9)	**0.013665**	0.129814
frost	10	32.4812±0.6698	32.9706±0.6955	0.4894 [–0.2059, 1.1847]	1.5923(9)	0.145790	0.595361
gaussian_blur	10	36.8574±0.7569	36.4956±0.7931	–0.3618 [–1.2406, 0.5170]	–0.9313(9)	0.376017	0.595361
gaussian_noise	10	38.3990±0.8211	38.6140±0.7924	0.2150 [–0.5355, 0.9655]	0.6480(9)	0.533152	0.779222
glass_blur	10	40.9016±0.3473	40.5698±0.4144	–0.3318 [–0.6895, 0.0259]	–2.0984(9)	0.065290	0.413503
impulse_noise	10	31.5040±0.7879	31.9046±1.2577	0.4006 [–0.5401, 1.3413]	0.9634(9)	0.360531	0.595361
jpeg_compression	10	43.0866±0.3449	43.1608±0.5395	0.0742 [–0.3991, 0.5475]	0.3546(9)	0.731041	0.782240
motion_blur	10	35.6704±0.8610	35.2446±0.8476	–0.4258 [–1.2724, 0.4208]	–1.1378(9)	0.284583	0.595361
pixelate	10	44.4638±0.3772	44.4046±0.6278	–0.0592 [–0.6756, 0.5572]	–0.2172(9)	0.832860	0.832860
saturate	10	29.3134±0.4581	29.6556±0.5910	0.3422 [–0.2391, 0.9235]	1.3318(9)	0.215673	0.595361
shot_noise	10	40.5094±0.7147	40.6094±0.5899	0.1000 [–0.5638, 0.7638]	0.3408(9)	0.741070	0.782240
snow	10	36.2324±0.5059	36.1604±0.3883	–0.0720 [–0.5149, 0.3709]	–0.3678(9)	0.721535	0.782240
spatter	10	39.1806±0.3955	38.9896±0.3984	–0.1910 [–0.6038, 0.2218]	–1.0466(9)	0.322583	0.595361
speckle_noise	10	40.2052±0.6375	40.3058±0.5620	0.1006 [–0.5019, 0.7031]	0.3777(9)	0.714379	0.782240
zoom_blur	10	37.6016±0.8617	37.1006±0.7064	–0.5010 [–1.3954, 0.3934]	–1.2671(9)	0.236925	0.595361

Note. Diff = mean(Neural-aligned–baseline). p values are two-tailed paired t-tests across seeds. p_{FDR} denotes Benjamini–Hochberg adjusted p across corruption types.

Table S4: Paired t-tests comparing DCCA (multi-stage neural alignment) vs. baseline average robustness accuracies across 10 seeds for each corruption type. Reported are mean±SD per condition, mean difference (Neural-aligned–baseline) with 95% CI, t(df), two-tailed p and Benjamini–Hochberg FDR-adjusted p.

Corruption	n	Baseline (Mean±SD)	Neural-aligned (Mean±SD)	Diff [95% CI]	t(df)	p	p_{FDR}
brightness	10	38.5704±0.6132	39.5776±0.7255	1.0072 [0.4081, 1.6063]	3.8033(9)	**0.004196**	0.079727
contrast	10	24.7328±0.5121	25.6766±1.6601	0.9438 [–0.1864, 2.0740]	1.8891(9)	0.091473	0.289664
defocus_blur	10	39.3360±0.6529	39.2586±0.5970	–0.0774 [–0.7442, 0.5894]	–0.2626(9)	0.798797	0.843174
elastic_transform	10	39.6832±0.6459	39.5950±0.5164	–0.0882 [–0.7107, 0.5343]	–0.3205(9)	0.755903	0.843174
fog	10	31.1314±0.3560	31.5760±1.2716	0.4446 [–0.5171, 1.4063]	1.0459(9)	0.322902	0.613514
frost	10	32.4812±0.6698	33.1540±1.0103	0.6728 [–0.0565, 1.4021]	2.0868(9)	0.066525	0.252796
gaussian_blur	10	36.8574±0.7569	36.5736±0.6832	–0.2838 [–0.9691, 0.4015]	–0.9369(9)	0.373285	0.618043
gaussian_noise	10	38.3990±0.8211	38.4184±0.8426	0.0194 [–0.6653, 0.7041]	0.0641(9)	0.950296	0.950296
glass_blur	10	40.9016±0.3473	40.6634±0.5750	–0.2382 [–0.7267, 0.2503]	–1.1031(9)	0.298625	0.613514
impulse_noise	10	31.5040±0.7879	31.2658±1.0552	–0.2382 [–0.8910, 0.4146]	–0.8254(9)	0.430464	0.618043
jpeg_compression	10	43.0866±0.3449	43.5836±0.4470	0.4970 [0.1103, 0.8837]	2.9072(9)	**0.017391**	0.089861
motion_blur	10	35.6704±0.8610	35.4586±0.5651	–0.2118 [–0.8910, 0.4674]	–0.7054(9)	0.498387	0.618043
pixelate	10	44.4638±0.3772	45.1338±0.4907	0.6700 [0.1392, 1.2008]	2.8555(9)	**0.018918**	0.089861
saturate	10	29.3134±0.4581	30.1452±0.7946	0.8318 [0.2288, 1.4348]	3.1206(9)	**0.012306**	0.089861
shot_noise	10	40.5094±0.7147	40.6944±0.6872	0.1850 [–0.4012, 0.7712]	0.7139(9)	0.493363	0.618043
snow	10	36.2324±0.5059	36.5110±0.7842	0.2786 [–0.3222, 0.8794]	1.0490(9)	0.321534	0.613514
spatter	10	39.1806±0.3955	39.4424±0.5067	0.2618 [–0.1815, 0.7051]	1.3361(9)	0.214315	0.581713
speckle_noise	10	40.2052±0.6375	40.3568±0.6418	0.1516 [–0.3559, 0.6591]	0.6758(9)	0.516156	0.618043
zoom_blur	10	37.6016±0.8617	37.3654±0.6686	–0.2362 [–1.0352, 0.5628]	–0.6687(9)	0.520457	0.618043

Note. Diff = Neural-aligned–baseline. p values are two-tailed; p_{FDR} denotes Benjamini–Hochberg adjusted p across corruption types.

Table S5: Paired t-tests for V1 (trained with DCCA loss) comparing Neural-aligned vs. baseline average robustness accuracies across $n = 10$ seeds for each corruption type. Reported are mean±SD per condition, mean difference (Neural-aligned–baseline) with 95% CI, t(df), two-tailed p and Benjamini–Hochberg FDR-adjusted p. Bold indicates $p < 0.05$.

Corruption	n	Baseline (Mean±SD)	Neural-aligned (Mean±SD)	Diff [95% CI]	t(df)	p	p_{FDR}
brightness	10	38.6462±0.5986	39.1471±0.6626	0.5009 [–0.0845, 1.0863]	1.9732(9)	0.083927	0.797304
contrast	10	24.7260±0.5426	24.5396±1.6942	–0.1864 [–1.4793, 1.1064]	–0.3326(9)	0.748011	0.978287
defocus_blur	10	39.4056±0.6521	39.6207±0.7680	0.2151 [–0.4676, 0.8978]	0.7266(9)	0.488208	0.978287
elastic_transform	10	39.7553±0.6409	39.8258±0.5607	0.0704 [–0.5065, 0.6474]	0.2816(9)	0.785422	0.978287
fog	10	31.1211±0.3760	31.0216±1.2844	–0.0996 [–1.1262, 0.9270]	–0.2236(9)	0.828653	0.978287
frost	10	32.5962±0.5966	32.9709±0.9634	0.3747 [–0.4334, 1.1828]	1.0692(9)	0.316195	0.978287
gaussian_blur	10	36.9471±0.7443	36.9804±0.8359	0.0333 [–0.7059, 0.7725]	0.1040(9)	0.919739	0.978287
gaussian_noise	10	38.5307±0.7506	38.4524±0.7457	–0.0782 [–1.0480, 0.8916]	–0.1860(9)	0.857078	0.978287
glass_blur	10	40.9556±0.3209	40.9524±0.3658	–0.0031 [–0.3782, 0.3720]	–0.0191(9)	0.985207	0.985207
impulse_noise	10	31.6338±0.7134	31.4800±1.1455	–0.1538 [–1.3064, 0.9989]	–0.3077(9)	0.766203	0.978287
jpeg_compression	10	43.1267±0.3403	43.4362±0.5716	0.3096 [–0.2560, 0.8751]	1.2623(9)	0.242400	0.978287
motion_blur	10	35.7533±0.8699	35.6047±0.8091	–0.1487 [–0.9288, 0.6314]	–0.4395(9)	0.671954	0.978287
pixelate	10	44.4849±0.3938	44.9833±0.4363	0.4984 [–0.0582, 1.0551]	2.0649(9)	0.072808	0.797304
saturate	10	29.3553±0.4651	29.5427±0.6348	0.1873 [–0.3714, 0.7461]	0.7731(9)	0.461693	0.978287
shot_noise	10	40.6269±0.6475	40.6807±0.6441	0.0538 [–0.7767, 0.8842]	0.1493(9)	0.884987	0.978287
snow	10	36.2744±0.5177	36.5658±0.5281	0.2913 [–0.2808, 0.8635]	1.1742(9)	0.274093	0.978287
spatter	10	39.2236±0.3940	39.3004±0.4339	0.0769 [–0.5226, 0.6764]	0.2957(9)	0.774955	0.978287
speckle_noise	10	40.2982±0.5999	40.4760±0.6472	0.1778 [–0.6234, 0.9789]	0.5117(9)	0.622685	0.978287
zoom_blur	10	37.7191±0.8246	37.6898±0.7709	–0.0293 [–0.7428, 0.6841]	–0.0948(9)	0.926798	0.978287

Note. Diff = Neural-aligned–baseline. p values are two-tailed paired t-tests across seeds. p_{FDR} denotes Benjamini–Hochberg adjusted p across corruption types.

Table S6: Paired t-tests for V4 (trained with DCCA loss) comparing Neural-aligned vs. baseline average robustness accuracies across $n = 10$ seeds for each corruption type. Reported are mean±SD per condition, mean difference (Neural-aligned–baseline) with 95% CI, t(df), two-tailed p and Benjamini–Hochberg FDR-adjusted p. Bold indicates $p < 0.05$.

Corruption	n	Baseline (Mean±SD)	Neural-aligned (Mean±SD)	Diff [95% CI]	t(df)	p	p_{FDR}
brightness	10	38.6462±0.5986	39.0760±0.9191	0.4298 [–0.3748, 1.2343]	1.2318(9)	0.252996	0.990145
contrast	10	24.7260±0.5426	24.3742±1.1536	–0.3518 [–1.2011, 0.4976]	–0.9551(9)	0.367510	0.990145
defocus_blur	10	39.4056±0.6521	39.5898±0.8345	0.1842 [–0.5673, 0.9358]	0.5652(9)	0.587403	0.990145
elastic_transform	10	39.7553±0.6409	39.9391±0.6417	0.1838 [–0.3899, 0.7575]	0.7387(9)	0.481189	0.990145
fog	10	31.1211±0.3760	31.0480±1.0730	–0.0731 [–1.1134, 0.9672]	–0.1621(9)	0.875273	0.990145
frost	10	32.5962±0.5966	32.6689±1.1309	0.0727 [–0.7328, 0.8781]	0.2080(9)	0.840398	0.990145
gaussian_blur	10	36.9471±0.7443	36.9220±0.8794	–0.0251 [–0.8367, 0.7864]	–0.0714(9)	0.944868	0.990145
gaussian_noise	10	38.5307±0.7506	38.2260±0.8963	–0.3047 [–1.1705, 0.5612]	–0.8114(9)	0.440583	0.990145
glass_blur	10	40.9556±0.3209	41.1076±0.5280	0.1520 [–0.2367, 0.5407]	0.9018(9)	0.393512	0.990145
impulse_noise	10	31.6338±0.7134	31.0858±1.0457	–0.5480 [–1.4881, 0.3921]	–1.3442(9)	0.215759	0.990145
jpeg_compression	10	43.1267±0.3403	43.4498±0.5763	0.3231 [–0.1387, 0.7849]	1.6135(9)	0.145301	0.990145
motion_blur	10	35.7533±0.8699	35.5856±0.9681	–0.1678 [–0.9641, 0.6285]	–0.4859(9)	0.640092	0.990145
**pixelate**	10	44.4849±0.3938	45.0953±0.5346	0.6104 [0.1301, 1.0908]	2.9304(9)	**0.018987**	0.360752
saturate	10	29.3553±0.4651	29.3647±0.7365	0.0093 [–0.6036, 0.6223]	0.0351(9)	0.972850	0.990145
shot_noise	10	40.6269±0.6475	40.5524±0.7448	–0.0744 [–0.7941, 0.6452]	–0.2385(9)	0.817464	0.990145
snow	10	36.2744±0.5177	36.3227±0.8154	0.0482 [–0.6090, 0.7055]	0.1692(9)	0.869849	0.990145
spatter	10	39.2236±0.3940	39.2207±0.5457	–0.0029 [–0.5257, 0.5199]	–0.0127(9)	0.990145	0.990145
speckle_noise	10	40.2982±0.5999	40.2224±0.7278	–0.0758 [–0.7315, 0.5799]	–0.2665(9)	0.796600	0.990145
zoom_blur	10	37.7191±0.8246	37.8089±0.9005	0.0898 [–0.8211, 1.0007]	0.2273(9)	0.825906	0.990145

Note. Diff = Neural-aligned–baseline. p values are two-tailed paired t-tests across seeds. p_{FDR} denotes Benjamini–Hochberg adjusted p across corruption types.

Table S7: Paired t-tests for IT (trained with DCCA loss) comparing Neural-aligned vs. baseline average robustness accuracies across $n = 10$ seeds for each corruption type. Reported are mean±SD per condition, mean difference (Neural-aligned–baseline) with 95% CI, t(df), two-tailed p and Benjamini–Hochberg FDR-adjusted p. Bold indicates $p < 0.05$.

Corruption	n	Baseline (Mean±SD)	Neural-aligned (Mean±SD)	Diff [95% CI]	t(df)	p	p_{FDR}
brightness	10	38.6462±0.5986	39.4462±0.6560	0.8000 [–0.0308, 1.6308]	2.2205(9)	0.057142	0.217139
contrast	10	24.7260±0.5426	24.7831±0.6035	0.0571 [–0.5050, 0.6192]	0.2343(9)	0.820649	0.921655
defocus_blur	10	39.4056±0.6521	39.6109±0.7487	0.2053 [–0.6218, 1.0325]	0.5724(9)	0.582746	0.851706
elastic_transform	10	39.7553±0.6409	39.8262±0.6853	0.0709 [–0.6958, 0.8375]	0.2132(9)	0.836485	0.921655
fog	10	31.1211±0.3760	31.4013±0.5736	0.2802 [–0.1604, 0.7208]	1.4666(9)	0.180671	0.429094
frost	10	32.5962±0.5966	32.5918±0.7590	–0.0044 [–0.7609, 0.7520]	–0.0135(9)	0.989522	0.989522
gaussian_blur	10	36.9471±0.7443	36.8811±0.7938	–0.0660 [–0.9892, 0.8572]	–0.1649(9)	0.873147	0.921655
gaussian_noise	10	38.5307±0.7506	37.8280±0.7697	–0.7027 [–1.5548, 0.1495]	–1.9014(9)	0.093759	0.296904
glass_blur	10	40.9556±0.3209	40.6747±0.5878	–0.2809 [–0.7743, 0.2125]	–1.3128(9)	0.225654	0.476380
impulse_noise	10	31.6338±0.7134	30.4276±0.8095	–1.2062 [–2.0889, –0.3235]	–3.1512(9)	**0.013573**	0.064549
jpeg_compression	10	43.1267±0.3403	43.7638±0.3995	0.6371 [0.2821, 0.9922]	4.1379(9)	**0.003263**	**0.045377**
motion_blur	10	35.7533±0.8699	35.5836±0.8329	–0.1698 [–1.0641, 0.7245]	–0.4378(9)	0.673122	0.913522
pixelate	10	44.4849±0.3938	45.3653±0.5777	0.8804 [0.3551, 1.4058]	3.8648(9)	**0.004777**	**0.045377**
saturate	10	29.3553±0.4651	30.0360±0.4694	0.6807 [0.1824, 1.1789]	3.1505(9)	**0.013589**	0.064549
shot_noise	10	40.6269±0.6475	40.3393±0.6841	–0.2876 [–1.0274, 0.4523]	–0.8963(9)	0.396269	0.684465
snow	10	36.2744±0.5177	35.8604±0.6573	–0.4140 [–1.0177, 0.1897]	–1.5815(9)	0.152430	0.413739
spatter	10	39.2236±0.3940	39.0851±0.5133	–0.1384 [–0.5646, 0.2877]	–0.7492(9)	0.475203	0.752404
speckle_noise	10	40.2982±0.5999	40.0153±0.6416	–0.2829 [–0.9420, 0.3762]	–0.9897(9)	0.351286	0.667443
zoom_blur	10	37.7191±0.8246	37.6440±0.8709	–0.0751 [–1.0774, 0.9272]	–0.1728(9)	0.867096	0.921655

Note. Diff = Neural-aligned–baseline. p values are two-tailed paired t-tests across seeds. Bold indicates raw significance ($p < 0.05$). p_{FDR} denotes Benjamini–Hochberg adjusted p across corruption types.

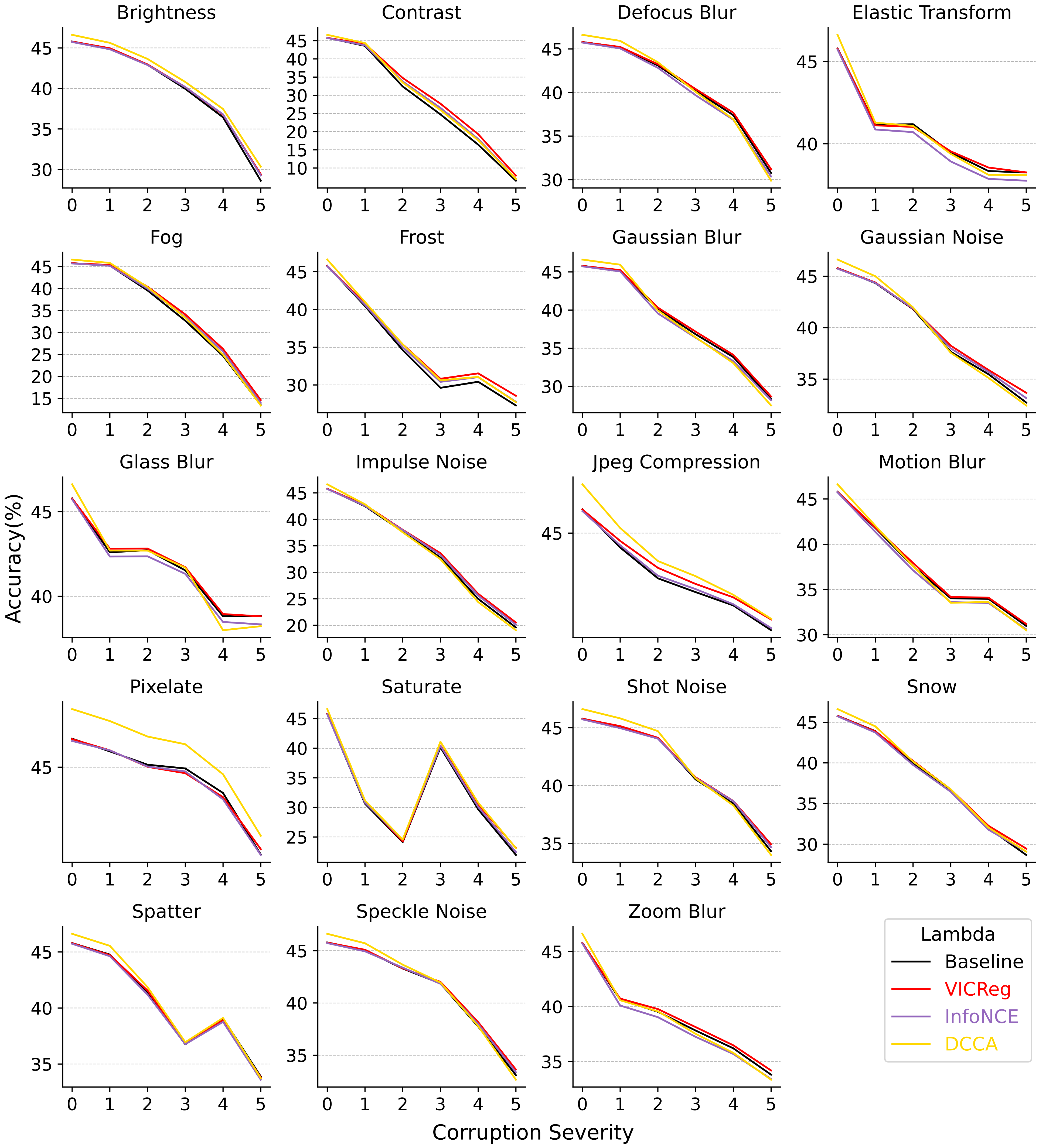

Brightness
Contrast
Defocus Blur
Elastic Transform
Fog
Frost
Gaussian Blur
Gaussian Noise
Glass Blur
Impulse Noise
Jpeg Compression
Motion Blur
Pixelate
Saturate
Shot Noise
Snow
Spatter
Speckle Noise
Zoom Blur
Accuracy(%)
Corruption Severity
Lambda
Baseline
VICReg
InfoNCE
DCCA

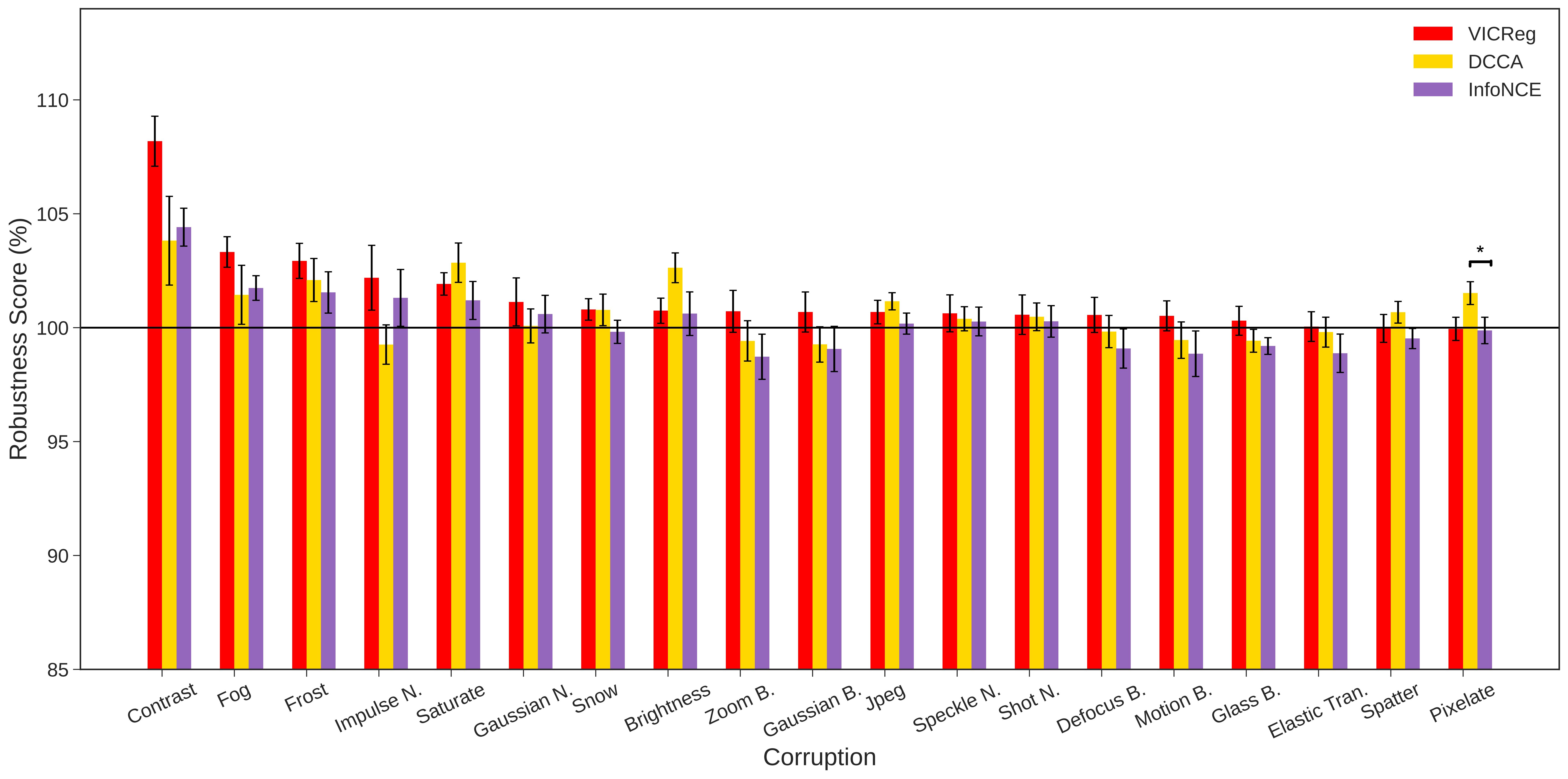

VICReg
DCCA
InfoNCE
Robustness Score (%)
110
105
100
95
90
85
*
Contrast
Fog
Frost
Impulse N.
Saturate
Gaussian N.
Snow
Brightness
Zoom B.
Gaussian B.
Jpeg
Speckle N.
Shot N.
Defocus B.
Motion B.
Glass B.
Elastic Tran.
Spatter
Pixelate
Corruption
